# Genetic Diversity and Candidate Selection Signatures in Hungarian and Romanian Carpathian Water Buffalo Inferred from Cross-Species SNP-Array Genotyping

**DOI:** 10.3390/ani16142120

**Published:** 2026-07-08

**Authors:** Szilvia Kusza, Putri Kusuma Astuti, Daniela Elena Ilie, Szilárd Pinnyey, Bettina Hegedűs, Husein Ohran, Zoltán Bagi, Dinu Gavojdian

**Affiliations:** 1 Centre for Agricultural Genomics and Biotechnology, University of Debrecen, Egyetem tér 1, 4032 Debrecen, Hungary; astuti@agr.unideb.hu (P.K.A.); hegedus.bettina@agr.unideb.hu (B.H.); bagiz@agr.unideb.hu (Z.B.); 2 Research and Development Station for Bovine, Bodrogului 32, 310059 Arad, Romania; danailie@animalsci-tm.ro; 3 Institute of Animal Sciences and Wildlife Management, University of Szeged, 6800 Hódmezővásárhely, Hungary; pinnyey.szilard@szte.hu; 4 Department of Physiology, University of Sarajevo, Zmaja od Bosne 90, 71000 Sarajevo, Bosnia and Herzegovina; husein.ohran@vfs.unsa.ba; 5 Research and Development Institute for Bovine, Balotesti, Bucuresti-Ploiesti km 21, 077015 Ilfov, Romania; gavojdian_dinu@animalsci-tm.ro

**Keywords:** *Bubalus bubalis*, Carpathian water buffalo, genetic diversity, population structure, cross-species SNP genotyping, runs of homozygosity, selection signatures, conservation genomics

## Abstract

The Carpathian water buffalo represents a locally adapted but under-characterized genetic group found in Central and Eastern Europe. Genome-wide information on its genetic diversity, population structure and potential adaptive variation remains limited, particularly for Hungarian and Romanian populations. In this study, we genotyped 263 water buffalo individuals from Hungary and Romania using the GeneSeek Genomic Profiler Bovine 100K SNP array to evaluate genetic diversity, the population structure, runs of homozygosity (ROH) and candidate genomic regions showing signatures of selection. After quality control, 214 Hungarian and 33 Romanian individuals and 6605 SNPs were retained for downstream analyses. Both populations showed moderate genetic diversity, with the Romanian population displaying higher minor allele frequency, observed heterozygosity and nucleotide diversity than the Hungarian population. In contrast, the Hungarian buffalo showed a higher burden of runs of homozygosity, including a larger proportion of long ROH segments, suggesting stronger recent autozygosity or a more restricted breeding structure. Principal component analysis and neighbor-joining phylogeny separated the two populations, whereas ADMIXTURE indicated shared ancestry and a within-population substructure rather than complete population-specific differentiation. The integration of standardized *F_ST_*, absolute allele-frequency differences and ROH islands identified six candidate regions under a positive signature of selection in each population. These regions harbored genes previously associated with immune response, reproduction, growth, milk production and thermotolerance in bovids. Functional enrichment was limited, with significant Gene Ontology terms detected only in the Hungarian candidate regions. Our results provide a regional genomic baseline for the future conservation and breeding management of Carpathian water buffalo. Given the use of a cross-species SNP array and unequal sample sizes, the candidate selection signals should be interpreted as hypothesis-generating and warrant validation using higher-density buffalo-specific genomic data.

## Simple Summary

The Carpathian water buffalo is a regional livestock resource in Central and Eastern Europe, but little is known about its genetic background. This study compared buffalo from Hungary and Romania using genome-wide marker data generated with a cattle genotyping array. The results show that both populations retain moderate genetic diversity. The Romanian animals had slightly higher diversity, whereas the Hungarian animals showed more long homozygous genome segments, which may indicate stronger recent relatedness or a more restricted breeding structure. The candidate regions for positive selection included genomic regions that may be associated with immune response, reproduction, growth, milk production and heat or cold tolerance. Because the marker array was designed for cattle and sample sizes differed between countries, the findings should be considered a first genomic baseline rather than final evidence of causal variants. The results can support future conservation planning and more detailed studies using buffalo-specific genomic tools.

## 1. Introduction

The domestic water buffalo (*Bubalus bubalis*) is an important livestock species contributing to milk, meat, draught power and rural livelihoods in diverse production environments [1,2,3]. Domestic buffalo are commonly classified into two major types, river buffalo and swamp buffalo, which differ in their geographic distribution, production orientation, cytogenetic characteristics and domestication history [4,5,6,7]. Although water buffalo are distributed across five continents and represent a global population exceeding 200 million animals, their genomic characterization remains less advanced than that of cattle, particularly for small regional populations outside the major Asian and Mediterranean production basins [1,7,8].

Locally adapted livestock populations are increasingly recognized as important reservoirs of genetic variation for future breeding, climate resilience and sustainable production systems. Conservation of such populations is particularly relevant under changing agro-ecological conditions, where traits related to robustness, disease resistance, grazing ability and adaptation to marginal environments may become increasingly valuable [9,10]. Recent Hungarian evidence from dairy cattle also highlights the importance of thermal resilience in Central European livestock systems. Baccouri et al. [11] showed that heat stress around insemination reduced insemination success and suggested genetic variability in heat-stress resilience. Although based on cattle rather than buffalo, these findings further support the relevance of conserving locally adapted ruminant genetic resources under increasingly variable thermal conditions. In the Carpathian Basin, water buffalo have historically been maintained as multipurpose animals in smallholder farming systems and wetland landscapes [12]. More recently, they have gained renewed attention for their potential role in habitat management, especially in wet and semi-natural grassland ecosystems, where buffalo grazing can contribute to vegetation control and biodiversity-oriented conservation management [13].

The Carpathian water buffalo represents a regional genetic resource maintained mainly in Hungary, Romania, and neighboring areas, except for the Bulgarian Murrah, which is considered a genetically divergent breed [14]. Remaining herds are often found in conservation settings, national parks and small private farms, reflecting both their cultural value and their vulnerability to demographic decline. Because small and fragmented populations are prone to genetic drift, loss of diversity and increased autozygosity, genome-wide monitoring is needed to support conservation-oriented breeding and to avoid unintentional erosion of locally adapted genetic backgrounds. Such information is also important for balancing productivity goals with the preservation of breed identity and adaptive potential.

Earlier marker-based studies characterized genetic relationships and diversity among Asian, Indian and Mediterranean buffalo populations using mitochondrial, Y-chromosomal or microsatellite markers [4,6,15,16]. Previous genomic studies have provided important insights into water buffalo diversity, domestication history and post-domestication migration routes at a global scale [7,17]. European buffalo populations, including animals from Germany, Bulgaria, Romania and Hungary, have also been investigated in a broader international context, showing that regional European herds may retain distinct genetic backgrounds while sharing ancestry with other river buffalo populations [18]. More recently, selection-footprint analyses in Bulgarian, Hungarian and Romanian buffalo identified candidate genomic regions and genes associated with milk production, reproduction, growth, immune response and adaptation [19]. However, detailed genomic analyses focusing specifically on Hungarian and Romanian Carpathian buffalo populations remain limited. Consequently, there is still insufficient information on their within-population diversity, between-population differentiation, runs of homozygosity (ROH) and candidate genomic regions potentially shaped by selection, especially given the common border and history that the two countries share.

Genome-wide characterization of buffalo populations is increasingly facilitated by single-nucleotide polymorphism (SNP) genotyping and sequencing technologies; however, buffalo-specific high-density genomic resources are still less widely available than those for cattle [8]. Cross-species SNP-array genotyping using bovine marker panels can therefore provide a practical screening approach for under-characterized bovid populations. Nevertheless, this approach is affected by marker transferability, ascertainment bias and potential limitations in genome annotation. Accordingly, results obtained from bovine SNP arrays in buffalo should be interpreted as broad population-genomic evidence rather than high-resolution causal inference. Comparative genomic studies across cattle and water buffalo support the value of such approaches for identifying broad patterns of population structure and domestication-related genomic variation, while also emphasizing the need for cautious interpretation and further validation [8,17,20].

Therefore, the objective of the present study was to characterize the genetic diversity, population structure, autozygosity patterns and candidate selection-signature regions in Hungarian and Romanian Carpathian water buffalo populations using cross-species SNP-array genotyping. Specifically, we estimated diversity parameters, linkage disequilibrium (LD) decay, runs of homozygosity, the principal component structure, phylogenetic relationships, ancestry patterns and population differentiation. By providing a regional genomic baseline, this study aims to support conservation planning, breeding management and future genomic monitoring of Carpathian water buffalo.

## 2. Materials and Methods

### 2.1. Samples and Populations

A total of 263 water buffalo (*Bubalus bubalis*) individuals were included in this study, comprising 230 Hungarian buffalo (Hu) and 33 Romanian buffalo (Ro). Hungarian samples were collected from three geographically distinct locations in Hungary: a private farm in Csanádpalota, southeastern Hungary, and two conservation herds maintained in Hortobágy National Park in eastern Hungary and Fertő-Hanság National Park in northwestern Hungary. Romanian samples were collected from the Arad and Oradea regions in western Romania. Approximately 30 hair follicles per animal were pulled from unrelated individuals and stored into Hair Card (Neogen, Lansing, MI, USA) to be transported to Neogen Europe Ltd. (Ayr, UK) for further laboratory analyses.

### 2.2. Genotyping Dataset Preparation and Quality Control

All samples were genotyped using the GeneSeek Genomic Profiler™ (GGP) Bovine 100K SNP array (Neogen Corp., Lansing, MI, USA). Because the array was originally developed for cattle, a substantial reduction in informative markers was expected due to cross-species ascertainment bias and differences in genome organization between cattle and buffalo. Therefore, all downstream analyses were interpreted in the context of moderate marker density and potential marker-transferability bias. Genotype data were converted to PLINK binary format using PLINK v1.9 [21]. The initial dataset contained 95,256 variants across 263 individuals with a genotyping rate of 85.6%. Genomic coordinates were assigned using the SNP annotation file provided by the genotyping service, which were based on the UMD3.1/UMD3.1.1 reference genome using --update-chr and --update-map. After mapping, a small fraction of variants lacked genomic coordinates (549 variants, 0.576%) and were excluded. Quality control was performed by removing individuals with more than 30% missing genotypes (--mind 0.3), excluding SNPs with more than 30% missing genotypes (--geno 0.3), filtering out rare variants with a minor allele frequency below 0.01 (--maf 0.01), and excluding markers showing strong deviation from Hardy–Weinberg equilibrium (--hwe 1e^−7^), resulting in 6605 high-quality autosomal SNPs and 247 individuals, after removing 16 Hungarian samples with high missingness rates, making the final population consist of 214 Hungarian and 33 Romanian buffalo. Relatively permissive missingness thresholds were applied to retain informative cross-species markers while excluding samples and SNPs with excessive missing data.

### 2.3. Population Genomic Analyses

Observed and expected heterozygosity (H_O_ and H_E_, respectively) were calculated with PLINK v. 1.9 [21]. The inbreeding coefficient of an individual (I) relative to the subpopulation (S) (*F_IS_*) and the global fixation index (*F_ST_*) were calculated with the VCFtools version 0.1.16 [22]. Genome-wide nucleotide diversity (π), linkage disequilibrium (LD) decay, and ROH patterns were evaluated separately in Hu and Ro buffalo populations using the quality-controlled autosomal SNP dataset. Given the SNP density, for the reliability of the results, the ROH analysis was intentionally conservative and targeted only long ROH (≥4 Mb). FROH was calculated for each individual as the sum of detected ROH lengths divided by the total autosomal genome length. Nucleotide diversity (π) was estimated in VCFtools using a sliding-window approach with a 50 kb window size. Genome-wide LD decay was assessed for each population in PLINK v. 1.9 [21] using pairwise r^2^ values between autosomal SNPs with a minor allele frequency ≥ 0.05. Pairwise SNP distances were grouped into 20 kb bins, and the mean r^2^ value per bin was calculated. To reduce bin-to-bin noise associated with the moderate-density SNP panel, LD decay was visualized using the mean r^2^ against physical distance and done with R’s ggplot2 version 4.0.3 package [23].

### 2.4. Population Structure

For population structure analyses, the dataset was subjected to LD pruning using --indep-pairwise 50 5 0.2, where 50 represents the SNP window size, 5 the step size in SNPs, and 0.2 the (r^2) threshold used to remove highly correlated markers, which retained 2935 independent SNPs. Principal component analysis (PCA) was done using PLINK v1.9 [21]. Unsupervised ADMIXTURE analyses were performed for K = 2–10 ancestral populations using --cv = 10. Visualizations were done with R’s ggplot2 package [23]. A neighbor-joining (NJ) phylogeny was constructed using --distance square 1-ibs, and the NJ tree was inferred from the distance matrix in R version 01.2+418 using the ape version 5.8-1 [24] and phangorn version 1.1.2 [25] packages.

### 2.5. Detection of Selection Signatures

Selection signatures were investigated using complementary cross-population and within-population approaches in both buffalo populations and performed on the unpruned QCed dataset comprising 6605 autosomal SNPs. Cross-population selection signals were assessed using Weir and Cockerham’s *F_ST_* as implemented in VCFtools [22] with a fixed-SNP sliding-window approach with 10 SNP windows and a 5 SNP step. Then the mean *F_ST_* value was calculated and standardized to obtain ZFST. Across the genome, 1248 windows were tested, with a mean physical span of 3.482 Mb (median = 3.308 Mb; range = 0.046–10.734 Mb), reflecting the relatively low marker density of the dataset. The absolute allele-frequency differences (ΔAF) between populations were estimated from population-specific allele counts obtained in PLINK using the same sliding-window and standardized as ZΔAF. Windows in the upper 5% tails of the ZFST and ZΔAF distributions were considered candidate regions showing strong population differentiation.

Within-population selection signals were assessed using ROH with the following parameters: a minimum of 8 consecutive SNPs per ROH (--homozyg-snp 8), a minimum ROH length of 4 Mb (--homozyg-kb 4000), a minimum SNP density of 1 SNP per 1000 kb (--homozyg-density 1000), a maximum gap of 2000 kb between consecutive SNPs (--homozyg-gap 2000), a sliding window of 8 SNPs (--homozyg-window-snp 8), allowing one heterozygous and two missing genotypes per window (--homozyg-window-het 1, --homozyg-window-missing 2), and a window threshold of 0.05 (--homozyg-window-threshold 0.05). This parameter was set to allow for detection of long homozygous regions while maintaining sufficient marker support under the reduced SNP density. For each autosomal SNP, the proportion of individuals in which that SNP was included within an ROH was calculated, and SNPs in the upper 5% tail of the ROH-incidence distribution were retained as empirical outliers, and adjacent outlier SNPs located within 1 Mb were merged into a single ROH island.

Given the moderate marker density, ROH analyses were restricted to relatively long segments and were used primarily to compare broad autozygosity patterns between populations rather than to infer fine-scale inbreeding history.

Genomic windows in the upper 5% of both ZFST and ZΔAF distributions were first retained as empirical outlier windows showing relatively high population differentiation and allele-frequency divergence, and these were then overlapped with population-specific ROH islands. Because the dataset was based on a moderate-density SNP panel, overlaps were evaluated using a ±500 kb tolerance threshold. Regions supported by all three signals were considered consensus candidate putative selection regions, while acknowledging that some population differences may also reflect genetic drift. This multi-signal overlap strategy was used to reduce the likelihood of false-positive interpretation. However, because empirical outlier thresholds do not directly estimate false-positive rates, these regions were interpreted as preliminary candidate signals requiring further validation. Visualization was made using the circlize package version 0.4.18 [26].

### 2.6. Gene Annotation and Enrichment Analyses

These putative selection regions then were annotated against the bovine UMD3.1/UMD3.1.1 reference (GCA_000003055.5). The online Kyoto Encyclopedia of Genes and Genomes (KEGG) pathway and Gene Ontology (GO) were performed using the DAVID online database [27], using *Bos taurus* as the genome background, with a significance level of a Bonferroni *p* value < 0.05.

## 3. Results

Altogether 263 animals were genotyped; however, after quality control, 214 Hungarian samples remained for further analyses. Although the number of SNPs retained after quality control was substantially reduced, the remaining markers were distributed across the autosomes and were therefore considered suitable for assessing broad population-level patterns. However, this may limit the resolution of analyses that depend strongly on dense and evenly spaced markers, particularly short ROH detection, fine-scale LD estimation, and precise localization of candidate selection regions.

Genetic diversity analysis (Table 1) showed that both Hu and Ro buffalo populations retained moderate levels of diversity, with the Ro population maintaining slightly higher genetic variability than the Hu population. Furthermore, the negative *F_IS_* estimates in both populations indicate heterozygote excess relative to Hardy–Weinberg expectations. However, because *F_IS_* and ROH capture different aspects of inbreeding and autozygosity (*F_IS_* reflects population-level deviations from expected heterozygosity across loci, whereas ROH reflects individual autozygosity through long homozygous chromosomal segments), these results should be interpreted together with the ROH-based estimates.

**Figure 1:**
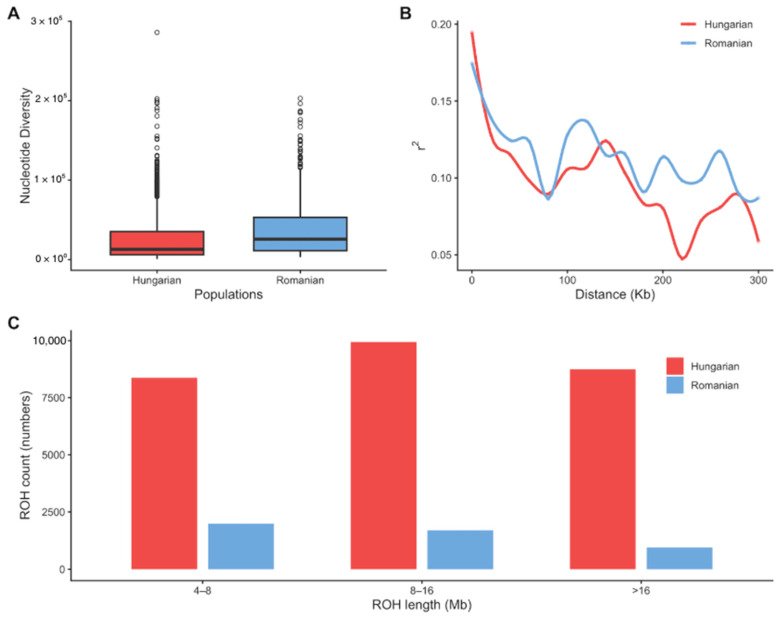
Population genomic diversity of Hungarian and Romanian buffalo. (**A**) Boxplot of genome-wide nucleotide diversity (π), (**B**) genome-wide average linkage disequilibrium decay, shown as mean r^2^ against physical distance, and (**C**) distribution of detected long ROH segments across length classes (4–8 Mb, 8–16 Mb, and >16 Mb).

Genome-wide nucleotide diversity showed that the Romanian population showed higher diversity than the Hungarian population (Figure 1A), suggesting higher overall genomic variability in the Ro population.

**Figure 2:**
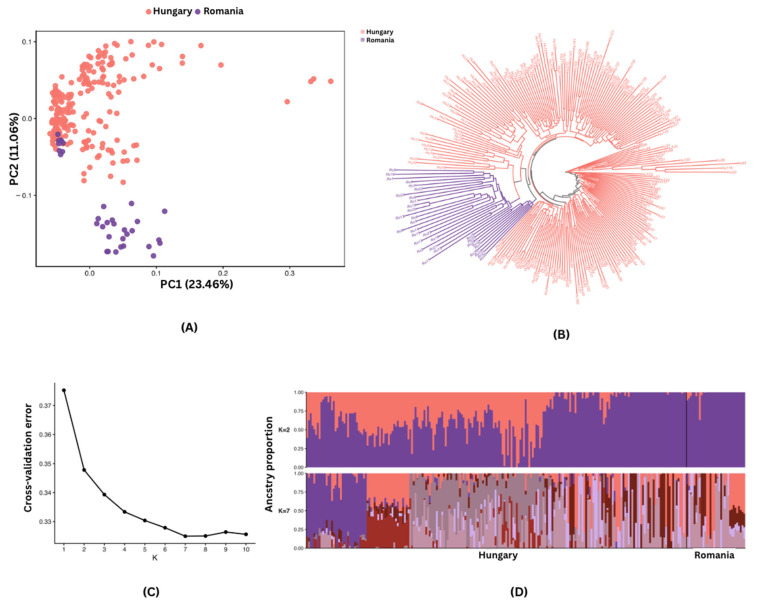
Genetic diversity analyses results. (**A**) Principal component analysis (PCA) plot, (**B**) neighbour-joining (NJ) phylogeny tree, and (**C**) ADMIXTURE’s cross-validation (CV) error with K from 1 to 10, and (**D**) plot distribution with K = 2 and K = 7 of Hungarian (N = 214) and Romanian (N = 33) buffalo.

The LD decay patterns shown in Figure 1B demonstrated a decline in r^2^ with increasing physical distance in both populations. Nevertheless, Romanian buffalo generally displayed higher r^2^ values over most distance classes, indicating slower LD decay compared to the Hu population. The Hu population showed a higher number of ROH segments across all classes (Figure 2C) compared to the Ro population. However, because of the unequal population sizes, these absolute counts should be interpreted with caution. When considered as proportions within each population, the 8–16 Mb class represented the largest fraction of detected long ROH in the Hungarian population (36.7%), while the Romanian population was dominated by 4–8 Mb ROH segments (42.8%). For the >16 Mb class, the Hu population has a higher proportion than the Ro population, accounting for 32.3% and 20.6%, respectively. Additionally, the ROH burden per individual to account for the unequal sample size showed that the Hu buffalo showed a higher mean number of detected ROH per animal than the Ro buffalo (72.21 vs. 48.42), together with a higher mean total ROH length (1179.36 vs. 757.44 Mb) and a higher mean FROH (0.48 vs. 0.31), indicating a greater burden of detectable long autozygous segments in the Hu population under the applied conservative ROH calling scheme. However, given the low-density SNP panel, these findings should be interpreted as detectable long homozygous segments rather than direct evidence of recent inbreeding.

The PCA plot showed meaningful separation between Hu and Ro buffalo populations clearly at PC2 (Figure 2A). The first two principal components explained 34.52% of the total genetic variance, with PC1 and PC2 explaining 23.46% and 11.06%, respectively. The Romanian buffalo formed a tighter cluster, while the Hungarian buffalo were more broadly dispersed, with relatively low overlap between some individuals from the two populations being observed. The NJ phylogenetic tree is also in agreement with the PCA; the Ro individuals formed a tighter cluster, whereas the Hu individuals were more widely dispersed, indicating greater genetic heterogeneity. This could also partly be because of the unequal sample sizes between the two populations. However, in terms of ancestry proportion analyses, the ADMIXTURE analysis showed unclear ancestry differentiation between the Hu and Ro buffalo, with the best CV error value at K = 7 (0.325) (Figure 2C). At K = 7, the Hungarian population displayed within-population genetic stratification and substructure, rather than complete, population-specific divergence from the Romanian population (Figure 2D).

**Figure 3:**
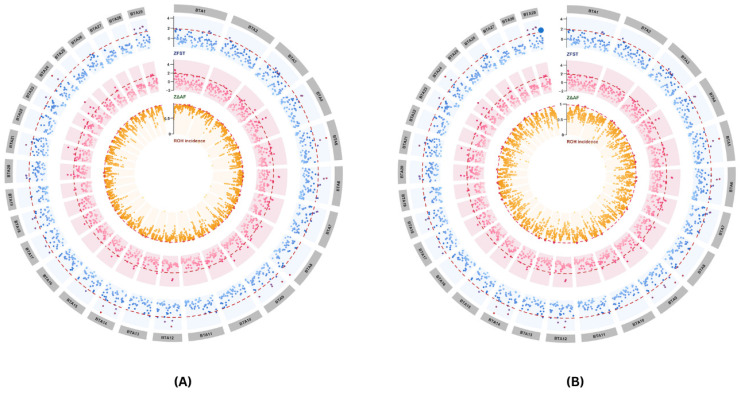
Circular Manhattan plot of genome-wide distribution of selection signatures in (**A**) Hungarian and (**B**) Romanian buffalo annotated against Bovine chromosome. The inner rings display ZFST, ZΔAF and ROH incidence.

For the Hu buffalo, 6 putative selection regions were detected based on the consensus of the three methods, which are located in chromosome BTA6, BTA7, BTA8, BTA14 and BTA21 (Figure 3A). Meanwhile, for the Ro buffalo, 6 regions were detected and located in chromosome 5, 14 and 18 (Figure 3B). These regions were found to harbor 287 genes and 86 genes (Table S1), respectively. No significantly enriched GO and KEGG pathways (Bonferroni *p* value < 0.05) were detected for the Ro buffalo population. Meanwhile, for Hu, only 5 GO pathways were significantly enriched (Bonferroni *p* value < 0.05): Protein maturation (GO-BP:0051604), Detection of chemical stimulus involved in sensory perception of smell (GO-BP:0050911), Granzyme-mediated programmed cell death signalling pathway (GO-BP:0140507), Intracellular membrane-bounded organelle (GO-CC:0043231) and Serine-type endopeptidase activity (GO-MF:0004252) (Table S2).

## 4. Discussion

Our study focused on the genetic characterization of the Carpathian buffalo for Hungarian and Romanian populations, defining their genetic structure and relationship to further elaborate their genetic potentials for future breed development and conservation. Amidst the emerging interest in buffalo genomics, our work complements recent selection-footprint analyses in Eastern European buffalo [19] by focusing specifically on Hungarian and Romanian Carpathian populations and by applying cross-species bovine SNP-array genotyping in this context. Cross-species genotyping could be a practical strategy for species with limited availability of species-specific genotyping platforms, although its efficiency depends on marker transferability and the evolutionary relatedness between the species. This point is further supported by the recent development of a buffalo-specific 100K SNP panel based on genotyping by target sequencing, which was designed from whole-genome sequence data and achieved a high call rate and genotype reproducibility, while producing population-genomic results highly consistent with whole-genome sequencing data [28]. In contrast to such buffalo-specific platforms, the use of a bovine SNP array in the present study should be regarded as an initial screening approach, useful for detecting broad population-genomic patterns but less suitable for fine-scale inference of causal variants. In addition to this, a limitation of our study is the sampling number disparity between the two populations that might influence the robustness of comparative analyses and the interpretation of between-population genetic differences. Furthermore, the two sampling sites from Romania (Arad and Bihor counties) share a common land border with Hungary; therefore, admixture could be suspected, given the relative recent introduction of Herdbooks for water buffalo in the two countries. Marker density and ascertainment bias may also affect ROH and LD estimates. In a recent comparison of buffalo genotyping platforms, the existing 90K Axiom array was shown to overestimate ROH and distort LD patterns in swamp buffalo because of the limited number of heterozygous sites captured by the array, whereas the newly developed 100K GBTS panel produced ROH and LD patterns more consistent with WGS [28]. Although the present study focuses on river-type Carpathian buffalo, these findings emphasize that ROH-based inferences from reduced or cross-species marker sets should be interpreted cautiously.

The number of informative SNPs identified in this study through cross-species genotyping appears comparable to previous works, although the low retained SNPs should be interpreted as broad genomic patterns rather than fine-scale genomic variation. Using the 100K GGP array, Burgos-Paz et al. [29] reported that only 11.7% of SNPs segregated uniformly across chromosomes in Colombian buffalo, while Shah et al. [30] found that only 1.7% of the 777K SNPs on the BovineHD BeadChip (13,150 SNPs) were sufficiently informative to define genomic diversity in Indian buffalo. The contrast with recently developed buffalo-specific panels is informative. Si et al. [28] reported that their 100K GBTS panel retained a high proportion of polymorphic markers in both river and swamp buffalo and provided population-structure, *F_ST_*, ROH and LD results that were highly consistent with WGS. Therefore, the reduced marker number in the present study most likely reflects cross-species ascertainment and marker-transferability limitations rather than a true lack of genomic variation in Carpathian buffalo. Buffalo and cattle share bovid ancestry yet underwent long evolutionary divergence, producing a species-specific genomic structure and organization. While chromosome numbers differ, both species share a similar autosomal fundamental number [31]. Furthermore, cross-species overlap in selection signals has been reported among domesticated Bovidae, supporting convergent domestication in which similar traits are targeted in comparable genomic regions across species [20], making the utilization of a bovine SNP chip in Carpathian buffalo biologically justified. However, it is crucial to recognize that river buffalo (2n = 50) and cattle (2n = 60) genomes, despite substantial autosome similarity, exhibit significant structural divergence.

The Hu population was found to be less genetically diverse compared to the Ro population, with a higher inbreeding sign. This pattern may reflect its husbandry structure in Hungary, where buffalo are predominantly maintained in small private herds for meat production and in national-park reserves, often without a specific breeding scheme. Meanwhile, in Romania, buffalo are kept for milk and meat production [32]. A similar high inbreeding level according to ROH was also reported by Noce et al. [18], which was potentially linked to the significant population decline in both countries for the past few decades.

In addition to this, as also reported by Noce et al. [18], the genetic differentiation analyses in this study suggested that the populations are genetically structured but only moderately differentiated between the two populations (global Hungarian vs. Romanian mean *F_ST_* = 0.03479). This is broadly consistent with the recent analysis of Bulgarian, Hungarian and Romanian buffalo, in which PCA and ADMIXTURE separated the Eastern European breeds while also indicating within-breed heterogeneity at higher K values [19]. Contrary to their observation of a more admixed Ro population, our results show that the Hu population has a greater within-group heterogeneity, although population-specific distinctiveness was not detected. The optimal K value should not be interpreted as the literal number of populations but represents the number of inferred ancestry components, which may reflect the within-population substructure, relatedness, and historical admixture. The moderate genetic differentiation between Hungarian and Romanian buffalo may reflect the historical geographic connectivity of buffalo populations across the Carpathian Basin, where animal movement and shared husbandry practices could have facilitated gene flow among regional herds. This interpretation is also compatible with whole-genome evidence from Sun et al. [33], who reported a deep genomic separation between river and swamp buffalo but also identified additional ancestry components within each buffalo type, including South Asian- and Italian-related components in river buffalo. Therefore, the moderate differentiation observed here between Hungarian and Romanian buffalo likely represents regional structuring within the river-buffalo genomic background rather than deep subspecies-level divergence. This interpretation is consistent with the broader genomic evidence showing that river buffalo populations generally display a weaker phylogeographic structure than swamp buffalo, despite substantial phenotypic and breed-level differentiation [7]. Similarly, a recent large-scale whole-genome study found that differentiation among populations within each buffalo type is relatively low compared with the deep divergence between river and swamp buffalo, supporting the view that regional river-buffalo populations may remain genetically connected despite local differentiation [34]. In addition, Neață and Vintilă [32] further explained that the second arrival of buffalo in Romania was by the Huns through the west part of the country during 11th–12th century. Similar occurrence was also observed in river buffalo from Egypt, Turkey, and Iran [17], which showed their genetic similarity and shared a common ancestry due to their geographical proximity that facilitated gene flow between them, given the theory of the Balkan route buffalo introduction into Eastern and Central Europe.

The only enriched GO-MF was Serine-type endopeptidase activity for the Hu population, which involved several genes that have been reported to have important roles in immunity in cattle: *AZU1* and *ELANE* are associated with the immune system [35]; *LOC508858* is linked to mastitis caused by infection with *S. aureus* [36]; *LOC100139881*, *GZMB* and *GZMM* were found to be related to antimicrobial peptide production [37,38,39]; and *PRSS57* and *PRTN3* are associated with biological functions in parasite infection [40]. In addition to this, production and reproduction-related genes have also been reported: *CFD* was linked to intramuscular fat production [41], *PCSK4* was associated with fertility and gestation [42], and *LOC786126* was specifically linked to bull reproduction [43]. For the Ro buffalo, amongst the 86 putative genes under positive selection, some have been reported to be key genes in buffalo production and adaptation: *SQLE*, *MYC*, *ELK3* were associated with milk production regulation [44,45], *SYCP3* was linked to spermatogenesis [46], *ALDH1L2* was linked to female buffalo reproduction [47], and *IGF1* was linked to muscle growth [48] and *HSBP1* to thermotolerance [49]. These broad functional categories agree with Saleh et al. [19], who reported overlapping selection regions in Eastern European buffalo containing candidate genes related to immune response, fertility, milk composition, milk production, reproduction, growth and adaptation, including *GAB2*, *LHCGR*, *FSHR*, *ST3GAL1* and *TMEM132C* in HU–RO comparisons. Similar trait categories have also been highlighted in broader buffalo genomic studies. Sun et al. [33] reported that genes under selection in river dairy buffalo were mainly related to heat stress and immunity, whereas Zhang et al. [50] showed that copy-number-variable regions differentiated between river and swamp buffalo and included genes associated with immunity, milk traits, endurance exercise and nervous-system functions. These results support the interpretation that the candidate regions detected in Carpathian buffalo may involve biologically relevant pathways related to immune response, production and environmental adaptation, although the present SNP-based analysis cannot capture all forms of genomic variation. Thermal stress environmental selection might have occurred, given the low winter temperatures registered in the Carpathian Mountains, significantly lower than in the domestication areas of the species. Moreover, the Romanian water buffalo, as an adaptative trait, in late autumn until mid-spring, grows a shaggy hair coat for cold-weather protection. Importantly, because the physical mapping in this study was performed against the bovine reference genome (UMD3.1), the reported chromosome coordinates represent bovine autosomes (BTAs) rather than native river buffalo chromosomes. Several genes located within the candidate regions have previously been associated with immune function, reproduction, production traits, or environmental adaptation in cattle or buffalo. However, these functional links should be interpreted cautiously, as the present study identifies genomic co-localization rather than direct causal relationships. The presence of these genes within candidate regions does not necessarily indicate that they are the causal targets of selection. Additionally, as the selection scan was based on 10-SNP windows in a sparse SNP panel, candidate regions should be interpreted as broad genomic intervals rather than narrow positional signals. Further validation using buffalo-specific genomic resources, particularly the recently developed 100K GBTS buffalo SNP panel or whole-genome sequencing, would be required to confirm these candidate regions and to clarify their role in local adaptation.

## 5. Conclusions

This study provides a genome-wide characterization of Hungarian and Romanian Carpathian water buffalo populations using cross-species SNP-array genotyping. Both populations retained moderate genetic diversity, but they differed in diversity indices, ROH patterns and population structure. The Hungarian population showed evidence of stronger autozygosity based on long ROH segments, whereas the Romanian population displayed slightly higher overall diversity estimates. Candidate selection-signature regions were detected in both populations and included genes previously linked to immunity, reproduction, growth, milk production and thermotolerance in bovids. Although these findings should be interpreted cautiously because of the moderate marker density, cross-species genotyping approach and unequal sample sizes, they provide an important baseline for conservation-oriented breeding and future genomic monitoring of Carpathian water buffalo. Future studies integrating buffalo-specific genomic resources with emerging genomic technologies will further improve the effectiveness of conservation-oriented breeding strategies [51].

## Figures and Tables

**Table 1:** Population genomic parameters calculated in the Hungarian and Romanian buffalo.

Population	N	Average MAF ^1^	*H_O_* ^2^	*H_E_* ^3^	*F_IS_* ^4^
Hungary	214	0.092 ± 0.115	0.149	0.146	−0.050
Romania	33	0.116 ± 0.141	0.183	0.148	−0.089

^1^ Minor allele frequency; ^2^ observed heterozygosity; ^3^ expected heterozygosity; ^4^ inbreeding coefficient of an individual relative to the subpopulation.

## Data Availability

The data generated from this study are publicly accessible via https://doi.org/10.5281/zenodo.20441541.

## Supplementary Materials

The following supporting information can be downloaded at: https://www.mdpi.com/article/10.3390/ani16142120/s1, Table S1: Putative genes under selection in Hungarian and Romanian buffalo, Table S2: The enriched GO term for Hungarian buffalo.

## Abbreviations

The following abbreviations are used in this manuscript:

BTA	*Bos taurus* autosome
CV	Cross-validation
DAVID	Database for Annotation, Visualization and Integrated Discovery
F_IS_	Inbreeding coefficient of an individual relative to the subpopulation
F_IT_	Inbreeding coefficient of an individual relative to the total population
F_ST_	Fixation index
GBTS	Genotyping by target sequencing
GCA	Genome assembly accession
GGP	GeneSeek Genomic Profiler
GO	Gene Ontology
GO-BP	Gene Ontology biological process
GO-CC	Gene Ontology cellular component
GO-MF	Gene Ontology molecular function
H_E_	Expected heterozygosity
H_O_	Observed heterozygosity
Hu	Hungarian buffalo population
KEGG	Kyoto Encyclopedia of Genes and Genomes
LD	Linkage disequilibrium
MAF	Minor allele frequency
NJ	Neighbor-joining
PCA	Principal component analysis
PLINK	Whole-genome association analysis toolset
QC	Quality control
Ro	Romanian buffalo population
ROH	Runs of homozygosity
SNP	Single-nucleotide polymorphism
UMD	University of Maryland bovine genome assembly
VCF	Variant call format
WGS	Whole-genome sequencing
ΔAF	Absolute allele-frequency difference
π	Nucleotide diversity
r^2^	Squared correlation coefficient
ZFST	Standardized F_ST_
ZΔAF	Standardized absolute allele-frequency difference
