# Supplementary material for: Genetic Diversity and Candidate Selection Signatures in Hungarian and Romanian Carpathian Water Buffalo Inferred from Cross-Species SNP-Array Genotyping
Source: Animals (Basel). 2026 Jul 8;16(14):2120. doi: 10.3390/ani16142120 (PMC13405233; doi:10.3390/ani16142120)
Supplement: Supplementary file 1 [file animals-16-02120-s001.zip › animals-4396063-supplementary.pdf]

Table S1. Putative genes under selection in Hungarian and Romanian buffalo.

Pop	Chr	Start Position (BP)	End Position (BP)	Overlapped Genes
HU	BTA14	52011701	55987263	*CSMD3*
	BTA14	54218992	59627973	*LOC107133125, TMEM74, KCNV1, ENY2, TRHR, EMC2, EIF3E, SYBU, EBAG9, PKHD1L1, NUDCD1, RSPO2, ANGPT1*
	BTA21	30995228	35609771	*PSMA4, ODF3L1, SNX33, IMP3, NEIL1, COMMD4, C21H15orf39, ULK3, CYP1A2, ISLR, ISLR2, LOC540321, LOC100139881, GZMB, LOC505326, LOC788565, LOC505658, LOC509956, LOC617313, LOC786126, LOC508858, LOC508646, SH2D7, CIB2, ACSBG1, DNAJA4, SKIC8, CRABP1, IREB2, HYKK, CHRNA5, CHRNA3, CHRNB4, UBE2Q2, NRG4, ETFA, RCN2, PSTPIP1, TSPAN3, HMG20A, CSPG4, SNUPN, PTPN9, MAN2C1, SCAMP5, RPP25, COX5A, MPI, CPLX3, LMAN1L, CSK, EDC3, CLK3, ARID3B, SEMA7A, LOC101903457, LOXL1, IDH3A, FBXO22, TMEM266, ISL2, PEAK1, SIN3A, PPCDC, SCAMP2, CYP1A1, CYP11A1, STRA6, PML, SCAPER, STXBP6*
	BTA6	36931767	40159975	*TIGD2, NAP1L5, PYURF, PIGY, MED28, DCAF16, HERC5, PKD2, MEPE, IBSP, LAP3, GPRIN3, FAM13A, HERC3, PPM1K, ABCG2, SPP1, FAM184B, LCORL, HERC6, NCAPG*
	BTA7	44022730	53046117	*PGBD2, OR2A83P, OR2AV1, OR2AV14, OR2AV2, OR2AV11, OR2BB12, OR2AZ4C, OR2AZ3B, OR2AZ1, C2CD4C, TPGS1, GZMM, BSG, FGF22, FSTL3, AZU1, PRTN3, CFD, LOC104972838, KISS1R, TMEM259, CNN2, POLR2E, GPX4, ATP5F1D, FAM174C, GAMT, DAZAP1, RPS15, LOC104972835, PCSK4, REEP6, LOC100337439, PLK5, MEX3D, MBD3, UQCR11, SOWAHA, GDF9, UQCRQ, LEAP2, ZCCHC10, CDKN2AIPNL, PITX1, TIFAB, NEUROG1, CXCL14, LOC518124, HNRNPA0, WNT8A, LOC101902951, GFRA3, FAM53C, REEP2, EGR1, MZB1, PROB1, IGIP, PLPP2, MIER2, THEG, SHC2, ODF3L2, MADCAM1, CDC34, HCN2, POLRMT, PRSS57, PALM, MISP, ELANE, PTBP1, MED16, R3HDM4, WDR18, GRIN3B, ARHGAP45, STK11, MIDN, EFNA2, PWWP3A, NDUFS7, C7H19orf25, ADAMTSL5, LOC107132633, KLF16, ABHD17A, SCAMP4, BTBD2, SHROOM1, C7H5orf15, VDAC1, TCF7, SKP1, CDKL3, UBE2B, JADE2, SAR1B, SEC24A, DDX46, C7H5orf24, TXNDC15, CATSPER3, MACROH2A1, IL9, LECT2, TGFBI, LOC104969184, MYOT, FAM13B, NME5, BRD8, CDC23, KIF20A, CDC25C, SLBP2, ETF1, LRRTM2, MATR3, PAIP2, SLC23A1, DNAJC18, ECSCR, CXXC5, PURA, SBNO2, APC2, ATP8B3, CSNK1G2, HSPA4, FSTL4, PPP2CA, CAMLG, PCBD2, SLC25A48, SMAD5, KLHL3, PKD2L2, HSPA9, CTNNA1, SIL1, SPATA24, PSD2, NRG2, ARID3A, AFF4, TRPC7, KDM3B, UBE2D2, SPOCK1*
	BTA8	94539339	100560959	*OR13F1, OR13C2E, OR13D2BP, OR13C2D, OR13F1B, OR13C2, OR13D2, OR13C2C, OR13C3, OR13D2C, OR13C8, OR13C1, TAL2, LOC104969469, ACTL7A, ABITRAM, SMC2, NIPSNAP3A, FKTN, TMEM38B, KLF4, ELP1, TMEM245, TOPORSL, ABCA1, SLC44A1, FSD1L, RAD23B, ACTL7B, CTNNAL1*
RO	BTA14	13328209	16863255	*MYC, TRIB1, WASHC5, SQLE, LRATD2, NSMCE2, ZNF572*
	BTA18	7043672	10193801	*DYNLRB2, MPHOSPH6, CENPN, ATMIN, C18H16orf46, GCSH, BCO1, SDR42E1, HSBP1, CDYL2, CMC2, LOC101905746, GAN, CMIP, HSD17B2, CDH13, PLCG2*
	BTA5	60587074	71321382	*SLC25A3, GARIN6, SYCP3, LOC101902154, PMCH, ASCL1, EID3, NOPCHAP1, BTBD11, CCDC38, AMDHD1, HAL, LTA4H, ELK3, IKBIP, ACTR6, SCYL2, SLC17A8, GAS2L3, ARL1, CHPT1,*

				NUP37, NT5DC3, HSP90B1, UQCC6, TDG, HCFC2, NFYB, ALDH1L2, CKAP4, TCP11L2, TMEM263, MTERF2, PWP1, CDK17, NEDD1, TMPO, APAF1, BLTP3B, NR1H4, SLC5A8, UTP20, SPIC, MYBPC1, GNPTAB, WASHC3, PARPBP, IGF1, PAH, STAB2, GLT8D2, TXNRD1, CHST11, SLC41A2, APPL2, NUAK1, POLR3B, CRY1, ANKS1B, ANO4, DRAM1, RFX4

Table S2. The enriched GO term for Hungarian buffalo.

GO	Term	Count	Bonferroni	Genes
BP	protein maturation	12	3.97E-07	*CFD, LOC505326, LOC508858, GZMM, LOC540321, LOC509956, LOC508646, LOC100139881, LOC786126, GZMB, PRSS57, AZU1*
BP	detection of chemical stimulus involved in sensory perception of smell	19	0.0000266	*OR2AZ1, OR2AV2, OR2AV1, OR2BB12, OR13C2C, OR2AZ4C, OR2AZ3B, OR13C8, OR13D2C, OR13C2D, OR13C2E, OR13F1B, OR13F1, OR13C1, OR13D2, OR13C3, OR2AV11, OR13C2, OR2AV14*
BP	granzyme-mediated programmed cell death signaling pathway	5	0.00893	*LOC505326, LOC508858, LOC508646, LOC786126, GZMB*
CC	intracellular membrane-bounded organelle	13	0.00725	*LOC505326, ABCA1, LOC508858, LOC505658, LOC509956, LOC508646, LOC100139881, GZMB, LOC540321, LOC786126, LOC617313, MISP, SYBU*
MF	serine-type endopeptidase activity	17	0.00000238	*CFD, LOC505326, LOC508858, LOC505658, LOC509956, LOC508646, LOC100139881, GZMB, PRSS57, AZU1, PCSK4, LOC540321, GZMM, LOC786126, LOC617313, PRTN3, ELANE*
